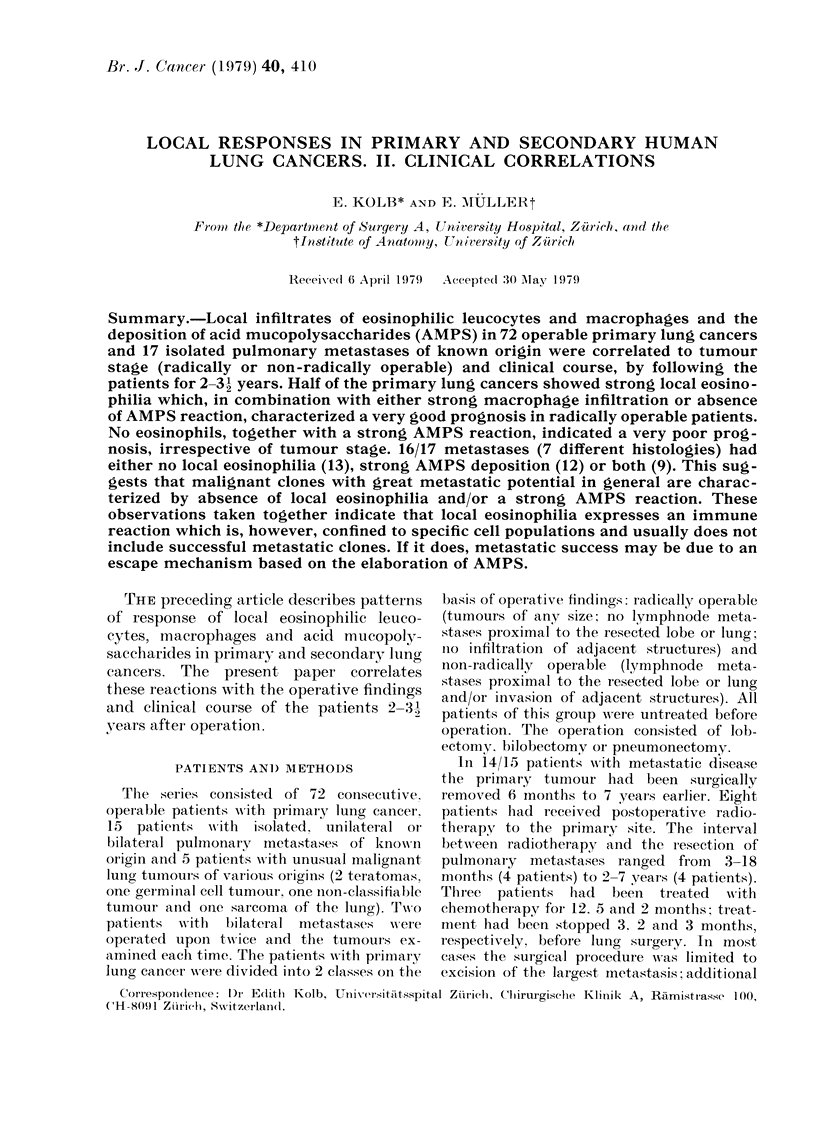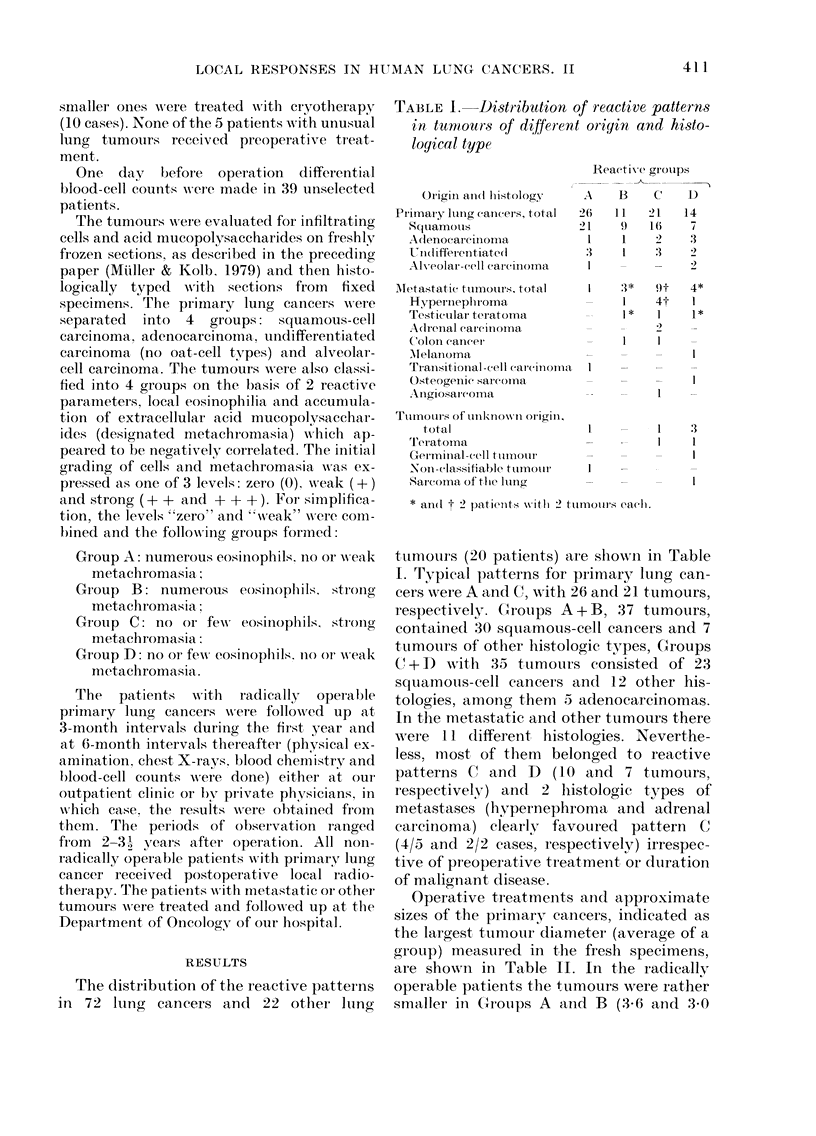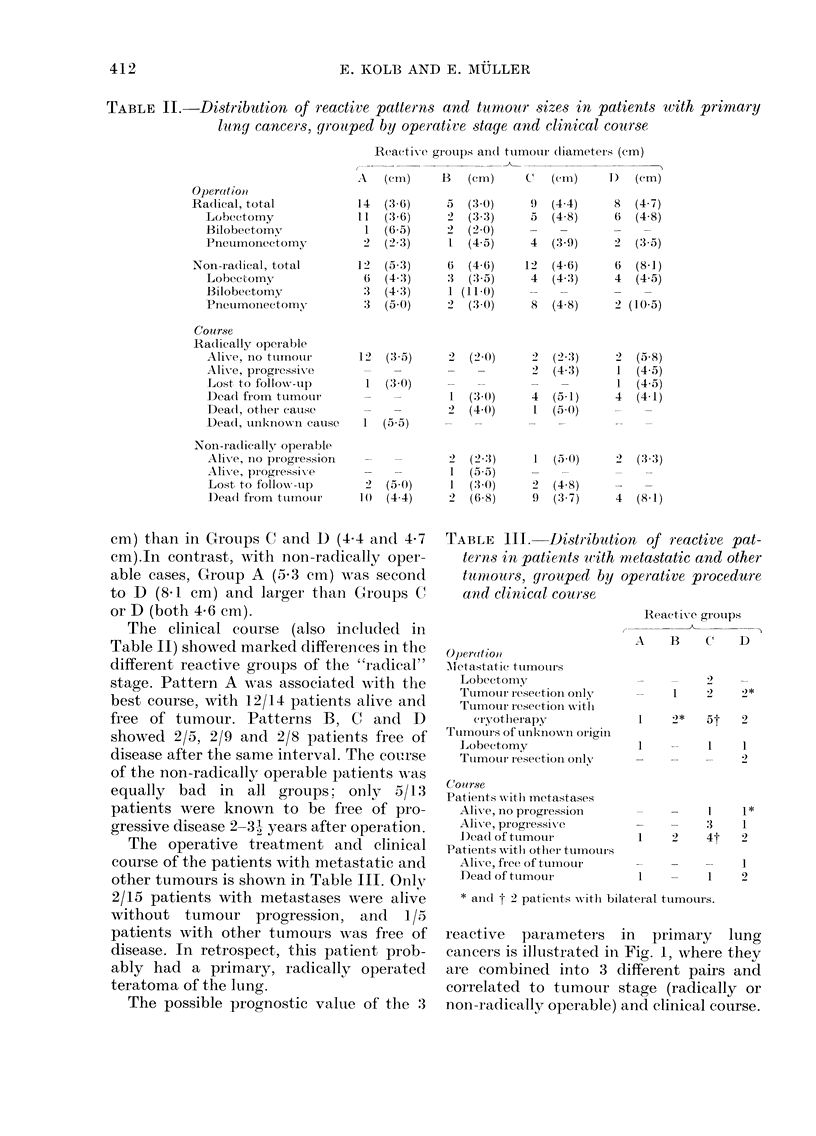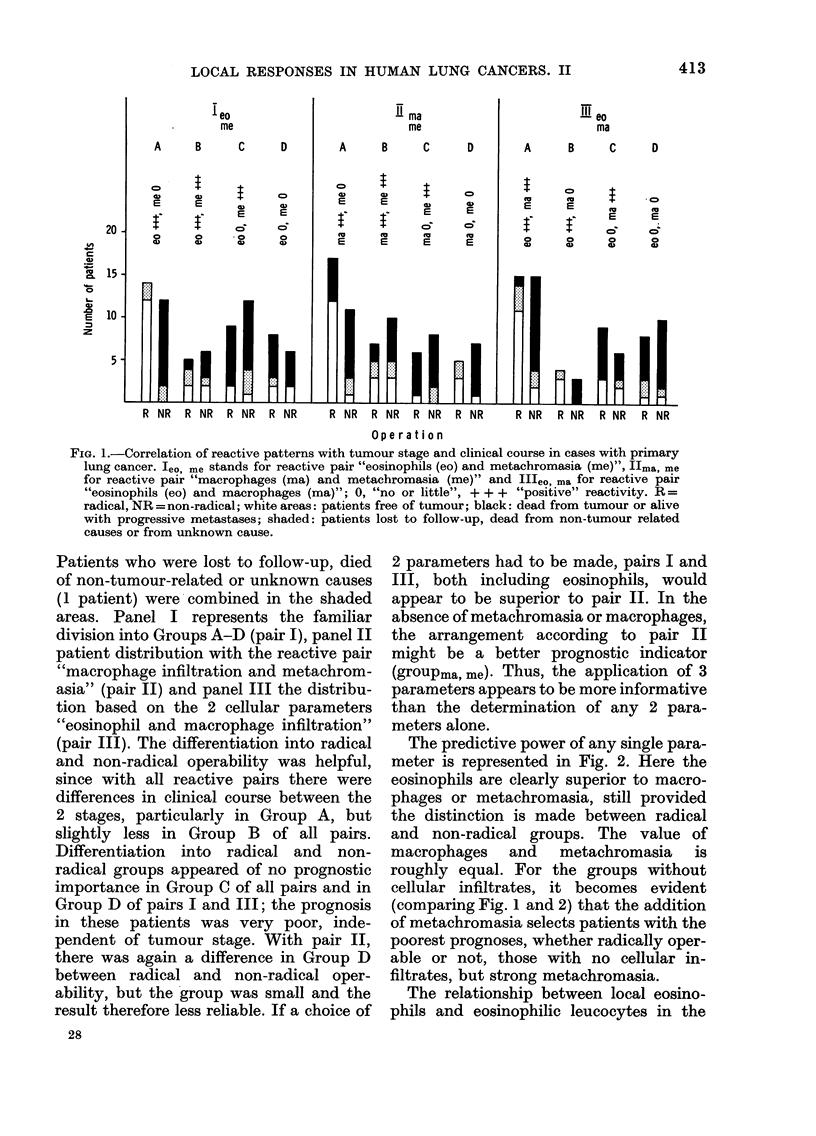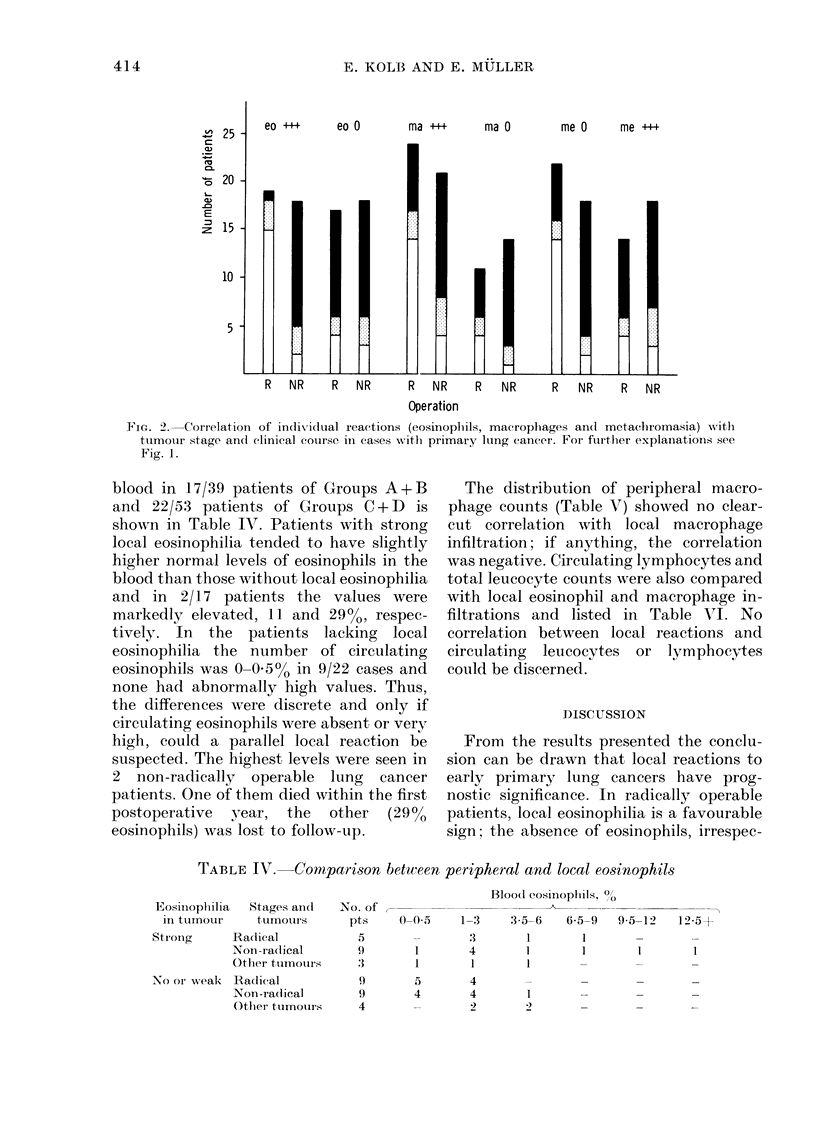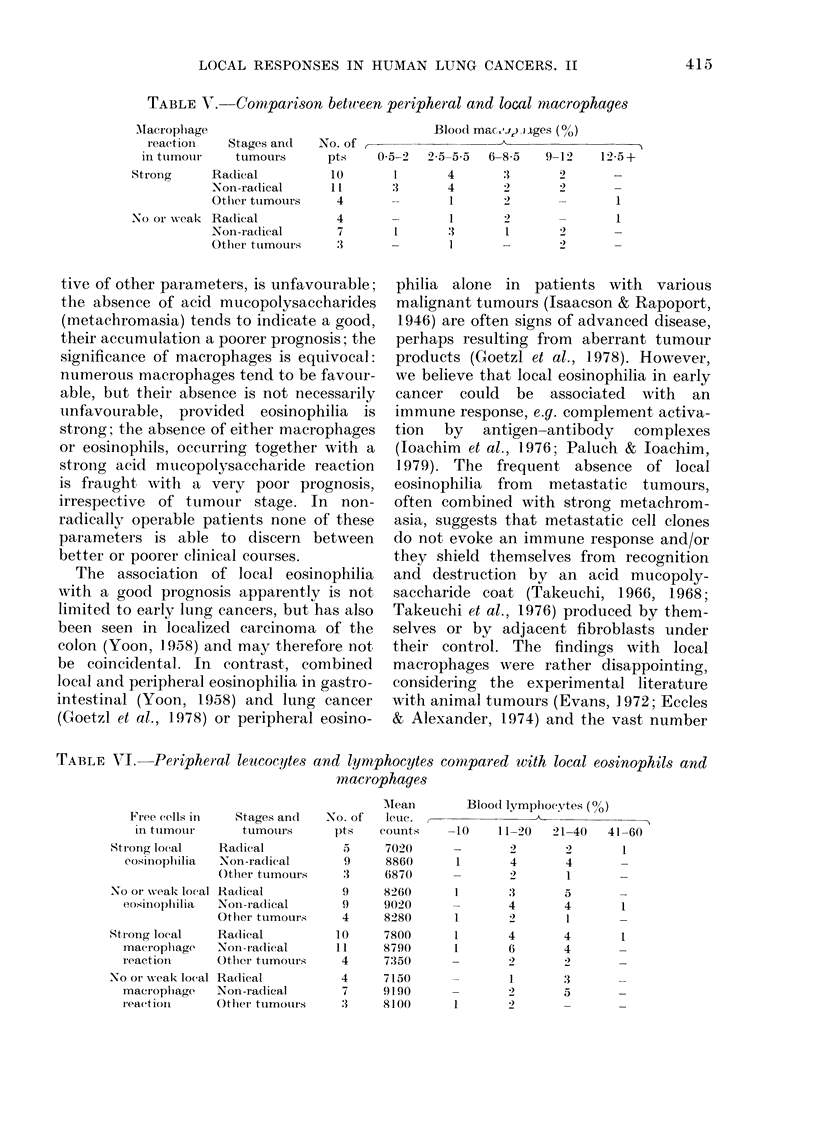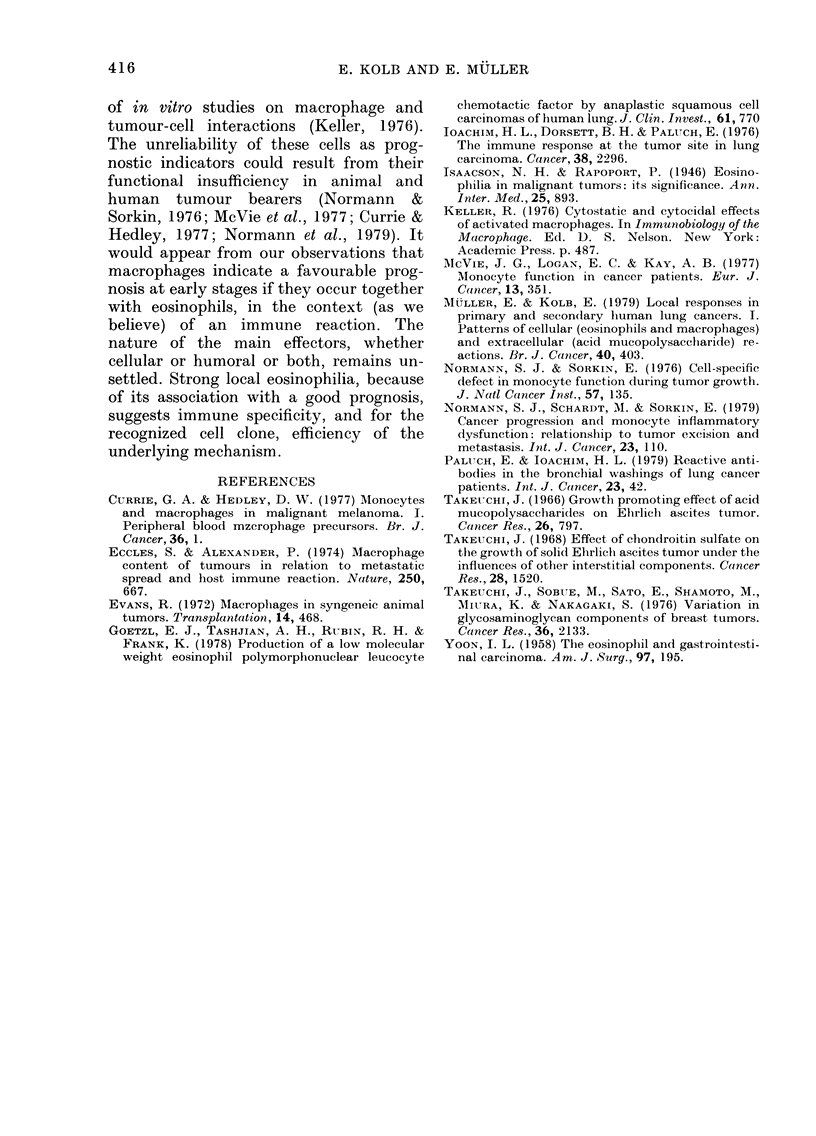# Local responses in primary and secondary human lung cancers. II. Clinical correlations.

**DOI:** 10.1038/bjc.1979.196

**Published:** 1979-09

**Authors:** E. Kolb, E. Müller

## Abstract

Local infiltrates of eosinophilic leucocytes and macrophages and the deposition of acid mucopolysaccharides (AMPS) in 72 operable primary lung cancers and 17 isolated pulmonary metastases of known origin were correlated to tumour stage (radically or non-radically operable) and clinical course, by following the patients for 2-3 1/2 years. Half of the primary lung cancers showed strong local eosinophilia which, in combination with either strong macrophage infiltration or absence of AMPS reaction, characterized a very good prognosis in radically operable patients. No eosinophils, together with a strong AMPS reaction, indicated a very poor prognosis, irrespective of tumour stage. 16/17 metastases (7 different histologies) had either no local eosinophilia (13), strong AMPS deposition (12) or both (9). This suggests that malignant clones with great metastatic potential in general are characterized by absence of local eosinophilia and/or a strong AMPS reaction. These observations taken together indicate that local eosinophilia expresses an immune reaction which is, houl metastatic clones. It if does, metastatic success may be due to an escape mechanism based on the elaboration of AMPS.


*Br. J. Cancer* (1979) **40**, 410

### LOCAL RESPONSES IN PRIMARY AND SECONDARY HUMAN LUNG CANCERS. II. CLINICAL CORRELATIONS

E. KOLB* AND E. MÜLLER†

*From the *Department of Surgery A, University Hospital, Zürich, and the †Institute of Anatomy, University of Zürich*

Received 6 April 1979 Accepted 30 May 1979

**Summary.—Local infiltrates of eosinophilic leucocytes and macrophages and the deposition of acid mucopolysaccharides (AMPS) in 72 operable primary lung cancers and 17 isolated pulmonary metastases of known origin were correlated to tumour stage (radically or non-radically operable) and clinical course, by following the patients for 2–3½ years. Half of the primary lung cancers showed strong local eosinophilia which, in combination with either strong macrophage infiltration or absence of AMPS reaction, characterized a very good prognosis in radically operable patients. No eosinophils, together with a strong AMPS reaction, indicated a very poor prognosis, irrespective of tumour stage. 16/17 metastases (7 different histologies) had either no local eosinophilia (13), strong AMPS deposition (12) or both (9). This suggests that malignant clones with great metastatic potential in general are characterized by absence of local eosinophilia and/or a strong AMPS reaction. These observations taken together indicate that local eosinophilia expresses an immune reaction which is, however, confined to specific cell populations and usually does not include successful metastatic clones. If it does, metastatic success may be due to an escape mechanism based on the elaboration of AMPS.**

THE preceding article describes patterns of response of local eosinophilic leucocytes, macrophages and acid mucopolysaccharides in primary and secondary lung cancers. The present paper correlates these reactions with the operative findings and clinical course of the patients 2–3½ years after operation.

### PATIENTS AND METHODS

The series consisted of 72 consecutive, operable patients with primary lung cancer, 15 patients with isolated, unilateral or bilateral pulmonary metastases of known origin and 5 patients with unusual malignant lung tumours of various origins (2 teratomas, one germinal cell tumour, one non-classifiable tumour and one sarcoma of the lung). Two patients with bilateral metastases were operated upon twice and the tumours examined each time. The patients with primary lung cancer were divided into 2 classes on the basis of operative findings: radically operable (tumours of any size; no lymphnode metastases proximal to the resected lobe or lung; no infiltration of adjacent structures) and non-radically operable (lymphnode metastases proximal to the resected lobe or lung and/or invasion of adjacent structures). All patients of this group were untreated before operation. The operation consisted of lobectomy, bilobectomy or pneumonectomy.

In 14/15 patients with metastatic disease the primary tumour had been surgically removed 6 months to 7 years earlier. Eight patients had received postoperative radiotherapy to the primary site. The interval between radiotherapy and the resection of pulmonary metastases ranged from 3–18 months (4 patients) to 2–7 years (4 patients). Three patients had been treated with chemotherapy for 12, 5 and 2 months; treatment had been stopped 3, 2 and 3 months, respectively, before lung surgery. In most cases the surgical procedure was limited to excision of the largest metastasis; additional

Correspondence: Dr Edith Kolb, Universitätsspital Zürich, Chirurgische Klinik A, Rämistrasse 100, CH-8091 Zürich, Switzerland.

smaller ones were treated with cryotherapy (10 cases). None of the 5 patients with unusual lung tumours received preoperative treatment.

One day before operation differential blood-cell counts were made in 39 unselected patients.

The tumours were evaluated for infiltrating cells and acid mucopolysaccharides on freshly frozen sections, as described in the preceding paper (Müller & Kolb, 1979) and then histologically typed with sections from fixed specimens. The primary lung cancers were separated into 4 groups: squamous-cell carcinoma, adenocarcinoma, undifferentiated carcinoma (no oat-cell types) and alveolar-cell carcinoma. The tumours were also classified into 4 groups on the basis of 2 reactive parameters, local eosinophilia and accumulation of extracellular acid mucopolysaccharides (designated metachromasia) which appeared to be negatively correlated. The initial grading of cells and metachromasia was expressed as one of 3 levels: zero (0), weak (+) and strong (++ and +++). For simplification, the levels "zero" and "weak" were combined and the following groups formed:

Group A: numerous eosinophils, no or weak metachromasia;
Group B: numerous eosinophils, strong metachromasia;
Group C: no or few eosinophils, strong metachromasia;
Group D: no or few eosinophils, no or weak metachromasia.

The patients with radically operable primary lung cancers were followed up at 3-month intervals during the first year and at 6-month intervals thereafter (physical examination, chest X-rays, blood chemistry and blood-cell counts were done) either at our outpatient clinic or by private physicians, in which case, the results were obtained from them. The periods of observation ranged from 2–3½ years after operation. All non-radically operable patients with primary lung cancer received postoperative local radiotherapy. The patients with metastatic or other tumours were treated and followed up at the Department of Oncology of our hospital.

### RESULTS

The distribution of the reactive patterns in 72 lung cancers and 22 other lung tumours (20 patients) are shown in Table I. Typical patterns for primary lung cancers were A and C, with 26 and 21 tumours, respectively. Groups A+B, 37 tumours, contained 30 squamous-cell cancers and 7 tumours of other histologic types, Groups C+D with 35 tumours consisted of 23 squamous-cell cancers and 12 other histologies, among them 5 adenocarcinomas. In the metastatic and other tumours there were 11 different histologies. Nevertheless, most of them belonged to reactive patterns C and D (10 and 7 tumours, respectively) and 2 histologic types of metastases (hypernephroma and adrenal carcinoma) clearly favoured pattern C (4/5 and 2/2 cases, respectively) irrespective of preoperative treatment or duration of malignant disease.

Operative treatments and approximate sizes of the primary cancers, indicated as the largest tumour diameter (average of a group) measured in the fresh specimens, are shown in Table II. In the radically operable patients the tumours were rather smaller in Groups A and B (3·6 and 3·0

TABLE I.—*Distribution of reactive patterns in tumours of different origin and histological type*

| | Reactive groups | | | |
|---|---|---|---|---|
| Origin and histology | A | B | C | D |
| Primary lung cancers, total | 26 | 11 | 21 | 14 |
| Squamous | 21 | 9 | 16 | 7 |
| Adenocarcinoma | 1 | 1 | 2 | 3 |
| Undifferentiated | 3 | 1 | 3 | 2 |
| Alveolar-cell carcinoma | 1 | – | – | 2 |
| Metastatic tumours, total | 1 | 3* | 9† | 4* |
| Hypernephroma | – | 1 | 4† | 1 |
| Testicular teratoma | – | 1* | 1 | 1* |
| Adrenal carcinoma | – | – | 2 | – |
| Colon cancer | – | 1 | 1 | – |
| Melanoma | – | – | – | 1 |
| Transitional-cell carcinoma | 1 | – | – | – |
| Osteogenic sarcoma | – | – | – | 1 |
| Angiosarcoma | – | – | 1 | – |
| Tumours of unknown origin, total | 1 | – | 1 | 3 |
| Teratoma | – | – | 1 | 1 |
| Germinal-cell tumour | – | – | – | 1 |
| Non-classifiable tumour | 1 | – | – | – |
| Sarcoma of the lung | – | – | – | 1 |

* and † 2 patients with 2 tumours each.

TABLE II.—*Distribution of reactive patterns and tumour sizes in patients with primary lung cancers, grouped by operative stage and clinical course*

| | Reactive groups and tumour diameters (cm) | | | | | | | |
|---|---|---|---|---|---|---|---|---|
| | A | (cm) | B | (cm) | C | (cm) | D | (cm) |
| *Operation* | | | | | | | | |
| Radical, total | 14 | (3·6) | 5 | (3·0) | 9 | (4·4) | 8 | (4·7) |
| Lobectomy | 11 | (3·6) | 2 | (3·3) | 5 | (4·8) | 6 | (4·8) |
| Bilobectomy | 1 | (6·5) | 2 | (2·0) | – | – | – | – |
| Pneumonectomy | 2 | (2·3) | 1 | (4·5) | 4 | (3·9) | 2 | (3·5) |
| Non-radical, total | 12 | (5·3) | 6 | (4·6) | 12 | (4·6) | 6 | (8·1) |
| Lobectomy | 6 | (4·3) | 3 | (3·5) | 4 | (4·3) | 4 | (4·5) |
| Bilobectomy | 3 | (4·3) | 1 | (11·0) | – | – | – | – |
| Pneumonectomy | 3 | (5·0) | 2 | (3·0) | 8 | (4·8) | 2 | (10·5) |
| *Course* | | | | | | | | |
| Radically operable | | | | | | | | |
| Alive, no tumour | 12 | (3·5) | 2 | (2·0) | 2 | (2·3) | 2 | (5·8) |
| Alive, progressive | – | – | – | – | 2 | (4·3) | 1 | (4·5) |
| Lost to follow-up | 1 | (3·0) | – | – | – | – | 1 | (4·5) |
| Dead from tumour | – | – | 1 | (3·0) | 4 | (5·1) | 4 | (4·1) |
| Dead, other cause | – | – | 2 | (4·0) | 1 | (5·0) | – | – |
| Dead, unknown cause | 1 | (5·5) | – | – | – | – | – | – |
| Non-radically operable | | | | | | | | |
| Alive, no progression | – | – | 2 | (2·3) | 1 | (5·0) | 2 | (3·3) |
| Alive, progressive | – | – | 1 | (5·5) | – | – | – | – |
| Lost to follow-up | 2 | (5·0) | 1 | (3·0) | 2 | (4·8) | – | – |
| Dead from tumour | 10 | (4·4) | 2 | (6·8) | 9 | (3·7) | 4 | (8·1) |

cm) than in Groups C and D (4·4 and 4·7 cm).In contrast, with non-radically operable cases, Group A (5·3 cm) was second to D (8·1 cm) and larger than Groups C or D (both 4·6 cm).

The clinical course (also included in Table II) showed marked differences in the different reactive groups of the "radical" stage. Pattern A was associated with the best course, with 12/14 patients alive and free of tumour. Patterns B, C and D showed 2/5, 2/9 and 2/8 patients free of disease after the same interval. The course of the non-radically operable patients was equally bad in all groups; only 5/13 patients were known to be free of progressive disease 2–3½ years after operation.

The operative treatment and clinical course of the patients with metastatic and other tumours is shown in Table III. Only 2/15 patients with metastases were alive without tumour progression, and 1/5 patients with other tumours was free of disease. In retrospect, this patient probably had a primary, radically operated teratoma of the lung.

The possible prognostic value of the 3 reactive parameters in primary lung cancers is illustrated in Fig. 1, where they are combined into 3 different pairs and correlated to tumour stage (radically or non-radically operable) and clinical course.

TABLE III.—*Distribution of reactive patterns in patients with metastatic and other tumours, grouped by operative procedure and clinical course*

| | Reactive groups | | | |
|---|---|---|---|---|
| | A | B | C | D |
| *Operation* | | | | |
| Metastatic tumours | | | | |
| Lobectomy | – | – | 2 | – |
| Tumour resection only | – | 1 | 2 | 2* |
| Tumour resection with cryotherapy | 1 | 2* | 5† | 2 |
| Tumours of unknown origin | | | | |
| Lobectomy | 1 | – | 1 | 1 |
| Tumour resection only | – | – | – | 2 |
| *Course* | | | | |
| Patients with metastases | | | | |
| Alive, no progression | – | – | 1 | 1* |
| Alive, progressive | – | – | 3 | 1 |
| Dead of tumour | 1 | 2 | 4† | 2 |
| Patients with other tumours | | | | |
| Alive, free of tumour | – | – | – | 1 |
| Dead of tumour | 1 | – | 1 | 2 |

* and † 2 patients with bilateral tumours.

FIG. 1.—Correlation of reactive patterns with tumour stage and clinical course in cases with primary lung cancer. $I_{eo,\ me}$ stands for reactive pair "eosinophils (eo) and metachromasia (me)", $II_{ma,\ me}$ for reactive pair "macrophages (ma) and metachromasia (me)" and $III_{eo,\ ma}$ for reactive pair "eosinophils (eo) and macrophages (ma)"; 0, "no or little", + + + "positive" reactivity. R = radical, NR = non-radical; white areas: patients free of tumour; black: dead from tumour or alive with progressive metastases; shaded: patients lost to follow-up, dead from non-tumour related causes or from unknown cause.

Patients who were lost to follow-up, died of non-tumour-related or unknown causes (1 patient) were combined in the shaded areas. Panel I represents the familiar division into Groups A–D (pair I), panel II patient distribution with the reactive pair "macrophage infiltration and metachromasia" (pair II) and panel III the distribution based on the 2 cellular parameters "eosinophil and macrophage infiltration" (pair III). The differentiation into radical and non-radical operability was helpful, since with all reactive pairs there were differences in clinical course between the 2 stages, particularly in Group A, but slightly less in Group B of all pairs. Differentiation into radical and non-radical groups appeared of no prognostic importance in Group C of all pairs and in Group D of pairs I and III; the prognosis in these patients was very poor, independent of tumour stage. With pair II, there was again a difference in Group D between radical and non-radical operability, but the group was small and the result therefore less reliable. If a choice of 2 parameters had to be made, pairs I and III, both including eosinophils, would appear to be superior to pair II. In the absence of metachromasia or macrophages, the arrangement according to pair II might be a better prognostic indicator ($group_{ma,\ me}$). Thus, the application of 3 parameters appears to be more informative than the determination of any 2 parameters alone.

The predictive power of any single parameter is represented in Fig. 2. Here the eosinophils are clearly superior to macrophages or metachromasia, still provided the distinction is made between radical and non-radical groups. The value of macrophages and metachromasia is roughly equal. For the groups without cellular infiltrates, it becomes evident (comparing Fig. 1 and 2) that the addition of metachromasia selects patients with the poorest prognoses, whether radically operable or not, those with no cellular infiltrates, but strong metachromasia.

The relationship between local eosinophils and eosinophilic leucocytes in the

FIG. 2.—Correlation of individual reactions (eosinophils, macrophages and metachromasia) with tumour stage and clinical course in cases with primary lung cancer. For further explanations see Fig. 1.

blood in 17/39 patients of Groups A+B and 22/53 patients of Groups C+D is shown in Table IV. Patients with strong local eosinophilia tended to have slightly higher normal levels of eosinophils in the blood than those without local eosinophilia and in 2/17 patients the values were markedly elevated, 11 and 29%, respectively. In the patients lacking local eosinophilia the number of circulating eosinophils was 0–0·5% in 9/22 cases and none had abnormally high values. Thus, the differences were discrete and only if circulating eosinophils were absent or very high, could a parallel local reaction be suspected. The highest levels were seen in 2 non-radically operable lung cancer patients. One of them died within the first postoperative year, the other (29% eosinophils) was lost to follow-up.

The distribution of peripheral macrophage counts (Table V) showed no clear-cut correlation with local macrophage infiltration; if anything, the correlation was negative. Circulating lymphocytes and total leucocyte counts were also compared with local eosinophil and macrophage infiltrations and listed in Table VI. No correlation between local reactions and circulating leucocytes or lymphocytes could be discerned.

### DISCUSSION

From the results presented the conclusion can be drawn that local reactions to early primary lung cancers have prognostic significance. In radically operable patients, local eosinophilia is a favourable sign; the absence of eosinophils, irrespec-

TABLE IV.—*Comparison between peripheral and local eosinophils*

| Eosinophilia in tumour | Stages and tumours | No. of pts | Blood eosinophils, % | | | | | |
|---|---|---|---|---|---|---|---|---|
| | | | 0–0·5 | 1–3 | 3·5–6 | 6·5–9 | 9·5–12 | 12·5+ |
| Strong | Radical | 5 | – | 3 | 1 | 1 | – | – |
| | Non-radical | 9 | 1 | 4 | 1 | 1 | 1 | 1 |
| | Other tumours | 3 | 1 | 1 | 1 | – | – | – |
| No or weak | Radical | 9 | 5 | 4 | – | – | – | – |
| | Non-radical | 9 | 4 | 4 | 1 | – | – | – |
| | Other tumours | 4 | – | 2 | 2 | – | – | – |

TABLE V.—*Comparison between peripheral and local macrophages*

| Macrophage reaction in tumour | Stages and tumours | No. of pts | Blood macrophages (%) | | | | |
|---|---|---|---|---|---|---|---|
| | | | 0·5–2 | 2·5–5·5 | 6–8·5 | 9–12 | 12·5+ |
| Strong | Radical | 10 | 1 | 4 | 3 | 2 | – |
| | Non-radical | 11 | 3 | 4 | 2 | 2 | – |
| | Other tumours | 4 | – | 1 | 2 | – | 1 |
| No or weak | Radical | 4 | – | 1 | 2 | – | 1 |
| | Non-radical | 7 | 1 | 3 | 1 | 2 | – |
| | Other tumours | 3 | – | 1 | – | 2 | – |

tive of other parameters, is unfavourable; the absence of acid mucopolysaccharides (metachromasia) tends to indicate a good, their accumulation a poorer prognosis; the significance of macrophages is equivocal: numerous macrophages tend to be favourable, but their absence is not necessarily unfavourable, provided eosinophilia is strong; the absence of either macrophages or eosinophils, occurring together with a strong acid mucopolysaccharide reaction is fraught with a very poor prognosis, irrespective of tumour stage. In non-radically operable patients none of these parameters is able to discern between better or poorer clinical courses.

The association of local eosinophilia with a good prognosis apparently is not limited to early lung cancers, but has also been seen in localized carcinoma of the colon (Yoon, 1958) and may therefore not be coincidental. In contrast, combined local and peripheral eosinophilia in gastro-intestinal (Yoon, 1958) and lung cancer (Goetzl *et al.*, 1978) or peripheral eosinophilia alone in patients with various malignant tumours (Isaacson & Rapoport, 1946) are often signs of advanced disease, perhaps resulting from aberrant tumour products (Goetzl *et al.*, 1978). However, we believe that local eosinophilia in early cancer could be associated with an immune response, *e.g.* complement activation by antigen–antibody complexes (Ioachim *et al.*, 1976; Paluch & Ioachim, 1979). The frequent absence of local eosinophilia from metastatic tumours, often combined with strong metachromasia, suggests that metastatic cell clones do not evoke an immune response and/or they shield themselves from recognition and destruction by an acid mucopolysaccharide coat (Takeuchi, 1966, 1968; Takeuchi *et al.*, 1976) produced by themselves or by adjacent fibroblasts under their control. The findings with local macrophages were rather disappointing, considering the experimental literature with animal tumours (Evans, 1972; Eccles & Alexander, 1974) and the vast number

TABLE VI.—*Peripheral leucocytes and lymphocytes compared with local eosinophils and macrophages*

| Free cells in tumour | Stages and tumours | No. of pts | Mean leuc. counts | Blood lymphocytes (%) | | | |
|---|---|---|---|---|---|---|---|
| | | | | –10 | 11–20 | 21–40 | 41–60 |
| Strong local eosinophilia | Radical | 5 | 7020 | – | 2 | 2 | 1 |
| | Non-radical | 9 | 8860 | 1 | 4 | 4 | – |
| | Other tumours | 3 | 6870 | – | 2 | 1 | – |
| No or weak local eosinophilia | Radical | 9 | 8260 | 1 | 3 | 5 | – |
| | Non-radical | 9 | 9020 | – | 4 | 4 | 1 |
| | Other tumours | 4 | 8280 | 1 | 2 | 1 | – |
| Strong local macrophage reaction | Radical | 10 | 7800 | 1 | 4 | 4 | 1 |
| | Non-radical | 11 | 8790 | 1 | 6 | 4 | – |
| | Other tumours | 4 | 7350 | – | 2 | 2 | – |
| No or weak local macrophage reaction | Radical | 4 | 7150 | – | 1 | 3 | – |
| | Non-radical | 7 | 9190 | – | 2 | 5 | – |
| | Other tumours | 3 | 8100 | 1 | 2 | – | – |

of *in vitro* studies on macrophage and tumour-cell interactions (Keller, 1976). The unreliability of these cells as prognostic indicators could result from their functional insufficiency in animal and human tumour bearers (Normann & Sorkin, 1976; McVie *et al.*, 1977; Currie & Hedley, 1977; Normann *et al.*, 1979). It would appear from our observations that macrophages indicate a favourable prognosis at early stages if they occur together with eosinophils, in the context (as we believe) of an immune reaction. The nature of the main effectors, whether cellular or humoral or both, remains unsettled. Strong local eosinophilia, because of its association with a good prognosis, suggests immune specificity, and for the recognized cell clone, efficiency of the underlying mechanism.

### REFERENCES

CURRIE, G. A. & HEDLEY, D. W. (1977) Monocytes and macrophages in malignant melanoma. I. Peripheral blood mzcrophage precursors. *Br. J. Cancer*, **36**, 1.

ECCLES, S. & ALEXANDER, P. (1974) Macrophage content of tumours in relation to metastatic spread and host immune reaction. *Nature*, **250**, 667.

EVANS, R. (1972) Macrophages in syngeneic animal tumors. *Transplantation*, **14**, 468.

GOETZL, E. J., TASHJIAN, A. H., RUBIN, R. H. & FRANK, K. (1978) Production of a low molecular weight eosinophil polymorphonuclear leucocyte chemotactic factor by anaplastic squamous cell carcinomas of human lung. *J. Clin. Invest.*, **61**, 770

IOACHIM, H. L., DORSETT, B. H. & PALUCH, E. (1976) The immune response at the tumor site in lung carcinoma. *Cancer*, **38**, 2296.

ISAACSON, N. H. & RAPOPORT, P. (1946) Eosinophilia in malignant tumors: its significance. *Ann. Inter. Med.*, **25**, 893.

KELLER, R. (1976) Cytostatic and cytocidal effects of activated macrophages. In *Immunobiology of the Macrophage*. Ed. D. S. Nelson. New York: Academic Press. p. 487.

MCVIE, J. G., LOGAN, E. C. & KAY, A. B. (1977) Monocyte function in cancer patients. *Eur. J. Cancer*, **13**, 351.

MÜLLER, E. & KOLB, E. (1979) Local responses in primary and secondary human lung cancers. I. Patterns of cellular (eosinophils and macrophages) and extracellular (acid mucopolysaccharide) reactions. *Br. J. Cancer*, **40**, 403.

NORMANN, S. J. & SORKIN, E. (1976) Cell-specific defect in monocyte function during tumor growth. *J. Natl Cancer Inst.*, **57**, 135.

NORMANN, S. J., SCHARDT, M. & SORKIN, E. (1979) Cancer progression and monocyte inflammatory dysfunction: relationship to tumor excision and metastasis. *Int. J. Cancer*, **23**, 110.

PALUCH, E. & IOACHIM, H. L. (1979) Reactive antibodies in the bronchial washings of lung cancer patients. *Int. J. Cancer*, **23**, 42.

TAKEUCHI, J. (1966) Growth promoting effect of acid mucopolysaccharides on Ehrlich ascites tumor. *Cancer Res.*, **26**, 797.

TAKEUCHI, J. (1968) Effect of chondroitin sulfate on the growth of solid Ehrlich ascites tumor under the influences of other interstitial components. *Cancer Res.*, **28**, 1520.

TAKEUCHI, J., SOBUE, M., SATO, E., SHAMOTO, M., MIURA, K. & NAKAGAKI, S. (1976) Variation in glycosaminoglycan components of breast tumors. *Cancer Res.*, **36**, 2133.

YOON, I. L. (1958) The eosinophil and gastrointestinal carcinoma. *Am. J. Surg.*, **97**, 195.